# Modeling Focal Epileptic Activity in the Wilson–Cowan Model with Depolarization Block

**DOI:** 10.1186/s13408-015-0019-4

**Published:** 2015-03-27

**Authors:** Hil G. E. Meijer, Tahra L. Eissa, Bert Kiewiet, Jeremy F. Neuman, Catherine A. Schevon, Ronald G. Emerson, Robert R. Goodman, Guy M. McKhann, Charles J. Marcuccilli, Andrew K. Tryba, Jack D. Cowan, Stephan A. van Gils, Wim van Drongelen

**Affiliations:** Department of Applied Mathematics, MIRA Institute for Biomedical Engineering and Technical Medicine, University of Twente, Postbus 217, Enschede, 7500AE The Netherlands; Department of Pediatrics, University of Chicago, KCBD 900 East 57th Street, Chicago, IL 60637 USA; Department of Neurology, Columbia University, 710 West 168th Street, New York, NY 10032 USA; Department of Neurological Surgery, Columbia University, 710 West 168th Street, New York, NY 10032 USA; Department of Physics, University of Chicago, 5720 South Ellis Avenue, Chicago, IL 60637 USA; Department of Mathematics, University of Chicago, 5734 South University Avenue, Chicago, IL 60637 USA

**Keywords:** Focal epilepsy, Activation function, Depolarization block, Bifurcation analysis

## Abstract

Measurements of neuronal signals during human seizure activity and evoked epileptic activity in experimental models suggest that, in these pathological states, the individual nerve cells experience an activity driven depolarization block, i.e. they saturate. We examined the effect of such a saturation in the Wilson–Cowan formalism by adapting the nonlinear activation function; we substituted the commonly applied sigmoid for a Gaussian function. We discuss experimental recordings during a seizure that support this substitution. Next we perform a bifurcation analysis on the Wilson–Cowan model with a Gaussian activation function. The main effect is an additional stable equilibrium with high excitatory and low inhibitory activity. Analysis of coupled local networks then shows that such high activity can stay localized or spread. Specifically, in a spatial continuum we show a wavefront with inhibition leading followed by excitatory activity. We relate our model simulations to observations of spreading activity during seizures.

**Electronic Supplementary Material:**

The online version of this article (doi:10.1186/s13408-015-0019-4) contains supplementary material 1.

## Introduction

Epilepsy is a neurological disease characterized by recurrent spontaneous seizures, i.e. episodes of abnormal excessive brain activity. Although epilepsy is one of the most prevalent neural diseases, affecting about 1% of the world population, the mechanisms governing seizure activity are not well understood and consequently treatment is unsuccessful for a significant fraction ($1/3$) of patients [1]. According to the clinical classification, epilepsy is a heterogeneous disease [2]. In spite of this heterogeneity in the pathology, there is also commonality between different seizure events suggesting that a variety of mechanisms may lead to a final common process, the seizure [3]. For example, in studies of brain slices it was demonstrated that seizure-like activity is characterized by spatial propagation, defined as failure of an inhibitory veto in neocortex [4], or failure of a dentate gate function in case of hippocampal driven events [5]. This shows that, in addition to a temporal evolution of a developing seizure, its spatial component at this mesoscopic level may be critically important. In fact, recently described micro-electrode array recordings in patients with epilepsy confirmed that propagation of neural activity occurs at a spatial scale below the size of a conventional cortical or scalp electroencephalogram (EEG) electrode [6]. At the microscopic level, intracellular measurements in human brain slices during evoked seizure activity show that neurons go into a depolarization block, i.e. they saturate, e.g. [7]. A recent report [8] describes an important role of the depolarization block in inhibitory cells in human cortical areas where seizures propagate, leading to the failed inhibition scenario described by [4]. In addition, it can be expected that under these high levels of activity, synaptic resources deplete, also contributing to a saturation effect. These data indicate that during high levels of seizure activity, hyperactive neurons may operate close to what can be described as an upper threshold of its input–output relationship. Such an epileptiform state would be in contrast to normal physiological operation of neuronal networks where the neurons operate around a lower activation threshold.

The goal of this study is to examine focal seizures propagating in cortex employing a modeling approach that includes details of the network under the EEG electrode. The tissue under the EEG electrode can be modeled by coupled neuronal populations [9–11]. Each population consists of an excitatory and inhibitory component. Many previous experimental [12] and theoretical [10, 13, 14] studies have shown that disinhibition can lead to traveling wave activity via blocking inhibition, assuming no synaptic inhibition or including a non-specific afferent affecting the inhibitory current. An important component in these studies is the sigmoidal activation function that describes the nonlinear relationship between the population’s input current reflected partially in the local field potential (LFP) and its output firing rate. In this study, we modified the equations to include a Gaussian firing rate function to reflect an upper-threshold phenomenon specific to the epileptiform network state. In Sect. 2 we present experimental evidence that such a function exists during seizures in the human cortex, and we incorporate this into the existing Wilson–Cowan formalism. Bifurcation analysis of single E-I neuron populations and a pair of coupled E-I populations is described in Sects. 3.1 and 3.2. In Sect. 3.3, we report simulation results showing the effect of the altered activation function on a network. In Sect. 4 we discuss the relevance of our new findings to our understanding of seizure propagation.

## Experimental Observations and Modeling

### Observations During Human Seizures

Both in vitro and in vivo electrophysiologic measurements suggest using an alternative to the commonly employed sigmoidal activation function in the Wilson–Cowan equations [9, 10] in our seizure model. One experimental component supporting this alternative function stems from single cell recordings obtained from human brain tissue resected from patients with drug-resistant epilepsy. During evoked seizures in cortical slices prepared from this brain tissue, single neurons show a strong paroxysmal depolarization, indicating an arrest of neuronal firing after high-level synaptic input exceeds an (upper) threshold; see e.g. [7].

**Fig. 1:**
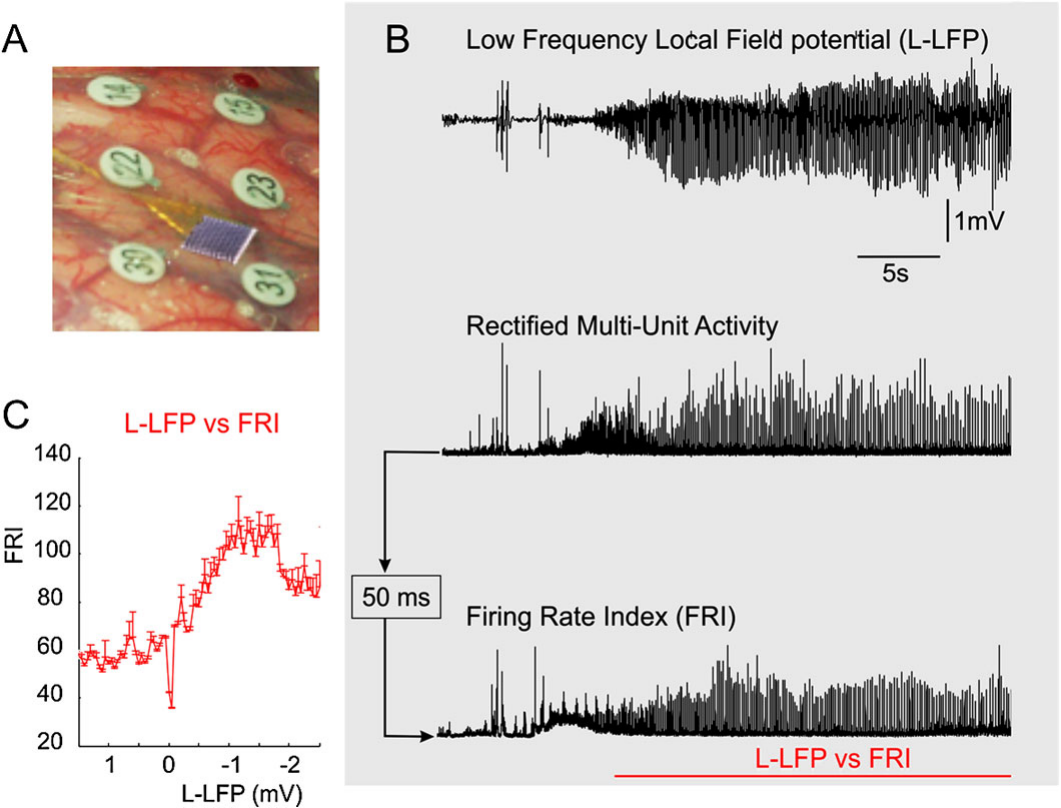
Experimental data supporting the use of a Gaussian population response function during human seizure activity. **A**: Recording setup depicting the multi-electrode array situated in between the standard electrocorticography electrodes numbered 22, 23, 30, and 31. **B**: Example recordings of the low-frequency component of the local field potential (2–50 Hz, L-LFP, *upper trace*), the rectified signal filtered for spikes (300–3000 Hz, *middle trace*), and the integrated version thereof, using a leaky integrator with a 50 ms time constant (*bottom trace*) generating a firing rate index (FRI) for the multi-unit spike activity. The relationship between L-LFP and FRI is plotted in panel **C**; the error bars indicate SEM values

A technique, recently approved for use in humans, allows application of micro-electrode recordings, during seizure activity [6]. Study participants consisted of adults with pharmaco-resistant focal epilepsy who underwent chronic invasive EEG studies to help identify the epileptogenic zone for subsequent removal. A 96, 4 mm × 4 mm, micro-electrode array (also known as Utah array) was implanted along with subdural electrodes with the goal of recording from seizure onset sites; see Fig. 1A. The study was approved by the Institutional Review Board of the Columbia University Medical Center, and informed consent was obtained from each patient prior to implantation. Signals from the micro-electrode array were acquired continuously at 30 kHz per channel (0.3 Hz–7.5 kHz bandpass, 16-bit precision, range ±8 mV). The reference was either subdural or epidural, chosen dynamically based on recording quality. See also [6] for details of study enrollment, surgical procedures and signal recording.

The signals in Fig. 1B were recorded from a single micro-electrode around seizure onset in a patient with intractable epilepsy. This in vivo recording shows the local field potential (LFP) that represents the weighted space-averaged electrical activity surrounding the electrode. The broadband signal from the micro-electrode can be filtered to examine its low-frequency component (L-LFP, 2–50 Hz) as well as the multi-unit spike activity (300–3000 Hz). We have examined the relationship between L-LFP and spike activity to study the population’s activation function. An index of the overall activity (firing rate index, FRI) was obtained by rectifying and integrating the spike traces (Fig. 1B, two bottom traces) [15]. The leaky integrator’s time constant employed here is 50 ms, which was chosen because it is close to the time constant of a cortical pyramidal cell [16]. We found that during seizure activity in focal areas where seizures are initiated, a plot of the FRI versus L-LFP is not a standard sigmoidal relationship, but rather is a mixture of sigmoid and Gaussian with a clear maximum; see Fig. 1C. To interpret this relationship properly, it should be noted that by convention, the L-LFP polarity is reversed, i.e. negative, relative to intracellular depolarization (positive). This relationship reflects contributions from inhibitory and excitatory neurons. We assume that the smaller neurons, especially the small inhibitory cells are saturated at high L-LFP levels which would explain the maximum.

The comparison between activation function and spike activity versus L-LFP is an approximation, based on a number of assumptions. First, the L-LFP is generated by multiple types of cellular current [17]. However, it is reasonable to assume that during the high levels of activity during seizures, the synaptic component will be the principal contributor [4, 6, 18]. In addition, a significant part of the non-synaptic sources of the L-LFP will be proportional to synaptic activity. In this context, it should be noted that such a relationship between synaptic activity and field potential has been the basis of many models of the electroencephalogram (EEG) as well, e.g. [19]. Next, we use the spike signal as a metric for network output while the multi-unit spike activity in a micro-electrode recording contains both input as well as output spikes of the local population. This is plausible since, due to geometry, the probability of picking up an output spike from an active neuron is much higher than recording from a thin afferent axon. Furthermore, if we assume the input spikes are proportional to the synaptic potentials they generate, they could only destroy the Gaussian-like result that we obtained in Fig. 1C. Another significant fact is that we only found Gaussian-like functions as in Fig. 1C within the epileptic core and not outside that area. This suggests that (inhibitory) cells reach depolarization block only within the core. Thus, although the relationship between L-LFP and multi-unit activity is not an exact measure of the population’s activation function, it is a reasonable proxy for it.

### Behavior of Single Cells During Seizures and in Biophysically Plausible Models

**Fig. 2:**
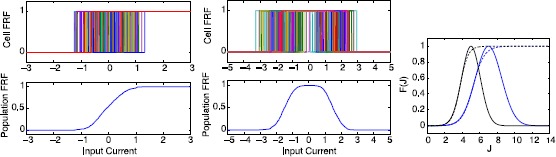
Constructing sigmoidal and Gaussian firing rate functions. *Left*: Heterogeneity in firing onset for individual cells leads to a sigmoidal population activation function. *Middle*: Including the effect of heterogeneous thresholds for depolarization block leads to a population activation function with a maximum. *Right*: The activation functions used in this paper Gaussian (*solid*) and sigmoid (*dashed*) for excitatory (*blue*) and inhibitory (*black*) populations

The activation function turns synaptic activity into a population firing rate, and is therefore also referred to as the firing rate function (FRF). Cells within a population have a slightly different firing threshold. In this simplified approach, we assume that the number of spikes does not depend on the input current, i.e. each cell has a Heaviside firing function. Summing all individual contributions, the jitter in thresholds leads to a sigmoidal function; see Fig. 2. In this regard, neurons do not only have a minimal value for the input current to spike, but also a maximal value where the membrane potential experiences a depolarization block. See, for instance, a dynamical systems explanation in [20], where it is called excitation block. Likewise, the precise critical value for the block will differ from cell to cell. Hence, for every cell, there is a finite range of input currents that results in spikes. Summing over the whole population leads to a Gaussian population activation function. This fundamental reasoning, based on the observation that the depolarization block occurring during evoked seizures represents an upper threshold for neuronal firing, also supports replacing a sigmoidal nonlinearity by a Gaussian-like activation function. There is some early work [21] supporting such a procedure.

The range of thresholds differs between cell types. For example, due to differences in the size, inhibitory neurons are activated by relatively small depolarizing inputs, whereas larger pyramidal neurons have a higher threshold. As inhibitory neurons are smaller, they have a propensity to reach depolarization block earlier than larger excitatory neurons during seizure activity. This is reflected in our choice of thresholds $E_{\theta}$, $I_{\theta}$, and standard deviations $E_{\mathrm{sd}}$, $I_{\mathrm{sd}}$; see also Fig. 2.

We noted above that there is a range of thresholds associated with both the excitatory and inhibitory populations. In the first Wilson–Cowan paper, it was assumed that these threshold distributions were either Poisson-like, or Gaussian. It then followed that the integrals of such curves would lead to an expression for the firing rate curves as the fraction of neurons receiving at least threshold excitation. In the distributions cited above, both integrals give rise to sigmoidal firing rate curves. Within this approach, it follows that a legitimate way of deriving a non-monotonic firing rate curve involves an additional threshold mechanism to express the effects of depolarization block.

### Modeling

**Fig. 3:**
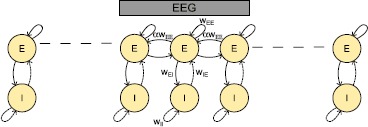
Overview of the local and global connections. Each excitatory population projects to the local inhibitory population and its neighboring excitatory population. Inhibitory populations only project to local excitatory populations. A model EEG output is defined as the average of the input currents to three excitatory populations

We model local microcircuits with an excitatory and an inhibitory population with weights for the connection strengths. We couple them to neighboring pairs via long range excitatory connections projecting to the excitatory population; see Fig. 3. The model is given by the following equations:

1 $$\begin{aligned} \tau_{X} X_{k}' =&-X_{k} + (1-X_{k})F_{X}(J_{X_{k}}), \end{aligned}$$

2 $$\begin{aligned} F_{X}(J_{X_{k}}) =& \exp \biggl(- \biggl(\frac{J_{X_{k}}-X_{\theta }}{X_{\mathrm{sd}}} \biggr)^{2} \biggr)-\exp \biggl(- \biggl(\frac{-X_{\theta }}{X_{\mathrm{sd}}} \biggr)^{2} \biggr), \end{aligned}$$

3 $$\begin{aligned} J_{E_{k}} =& w_{EE} E_{k} -w_{IE}I_{k}+ B + \alpha w_{EE} (E_{k+1}+E_{k-1}), \end{aligned}$$

4 $$\begin{aligned} J_{I_{k}} =& w_{EI} E_{k} -w_{II}I_{k}, \end{aligned}$$

where $X=E,I$ and $k=1,\ldots, N$. At the boundary the excitatory populations $E_{1}$ and $E_{N}$ get only input from $E_{2}$ and $E_{N-1}$, respectively. We use $\tau_{E}=\tau_{I}=1$, $w_{EE}=16$, $w_{EI}=18$, $w_{II}=3$, $w_{IE}=12$, $E_{\theta}=7$, $I_{\theta}=5$, $E_{\mathrm{sd}}=2.1$, and $I_{\mathrm{sd}}=1.5$. We use $B=3$, but vary this parameter throughout the paper. These values of the parameters are chosen as in previous modeling studies [9, 10, 22, 23], except for an increased value of $E_{\theta}$ and a different $E_{\mathrm{sd}}$. All bifurcation diagrams have been computed using matcont [24] and phase planes using [25]. For terminology on bifurcations we refer to [26, 27]. To compare our Gaussian FRF with the standard sigmoidal FRF, we also use

5 $$ F_{X}(J_{X_{k}}) = \bigl(1+\exp \bigl(-X_{s} (J_{X_{k}}-X_{\theta } ) \bigr) \bigr)^{-1}- \bigl(1+\exp (X_{s}X_{\theta} ) \bigr)^{-1}, $$

with $E_{\theta}=5.2516$, $E_{s}=1.5828$, $I_{\theta}=3.7512$ and $I_{s}=2.2201$. With these values, the Gaussian and sigmoid have the same slope at half activation. A model EEG is computed as the average of the synaptic inputs to three neighboring excitatory populations; see Fig. 3.

Finally, we also consider a spatially continuous model where we replace $X_{k}(t)$ by $X(y,t)$ where $y \in[0,L]$ and L=1000 μm. For this we replace the input currents by

6 $$ \begin{aligned} J_{E}(y,t) ={}& \lambda_{E} \int _{0}^{L} \bigl(w_{EE}e^{|y-z|/\sigma _{EE}}E(z,t) - w_{IE}e^{|y-z|/\sigma_{IE}}I(z,t) \bigr)\,dz \\ {}&+ B(y,t), \\ J_{I}(y,t) ={}& \lambda_{I} \int_{0}^{L} \bigl(w_{EI}e^{|y-z|/\sigma _{EI}}E(z,t) - w_{II}e^{|y-z|/\sigma_{II}}I(z,t) \bigr)\,dz, \end{aligned} $$

where $w_{EE} = 2.0$, $w_{IE}=1.65$, $w_{EI}=1.5$, $w_{II}=0.01$, $\sigma_{EE}=70\text{ μm}$, $\sigma_{IE}=90\text{ μm}$, $\sigma_{EI}=90\text{ μm}$, $\sigma_{II}=70\text{ μm}$, $E_{\theta}=18$, $E_{\mathrm{sd}}=6.7$, $I_{\theta }=10$, $I_{\mathrm{sd}}=3.2$. For the comparison to a sigmoid we use $E_{\theta }=12.41$, $E_{\mathrm{sd}}=2$, $I_{\theta}=7.33$, and $I_{\mathrm{sd}}=0.95$. These parameters are similar to the neural mass model used above, but scaled as we do not have normalized connectivity weights due to the finite domain. In this setup, tissue near the boundary receives less input. Furthermore, we set the densities of excitatory or inhibitory neurons in homogeneous and isotropic tissue as $\lambda_{E}=\lambda_{I}=1{\text{ μm}}^{-1}$. The input $B(y,t)$ consists of a constant background of 1 and a 100 μm wide, 10 ms square-wave pulse with amplitude 10.

## Bifurcation Analysis

### A Single E-I Pair

**Fig. 4:**
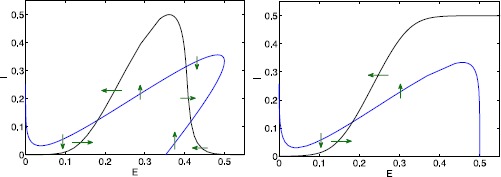
Phase planes for Gaussian (*left*) and sigmoid (*right*) activation function. Excitatory (*blue*) and inhibitory (*black*) nullclines and directions are shown for parameters as in Sect. 2 with $B=3$ and $w_{EI}=18$. Note the additional steady states for the Gaussian

For the reference values of the parameters we have done a phase-plane analysis; see Fig. 4. The excitatory nullcline for both Gaussian and sigmoid have a similar shape, although the Gaussian *E*-nullcline turns for high values of *E*. The *I*-nullclines differ more. For the sigmoidal activation function, the curve is monotonic, whereas for the Gaussian it has a hump. It has two more intersections with the *E*-nullcline yielding two more steady states, one saddle and one stable node, the latter with high excitatory activity and lower inhibitory activity. This additional stable equilibrium does not exist for the sigmoid. In this region, due to the depolarization block, the inhibitory cells reduce their output, while the excitatory cells generate sufficient recurrent excitation to maintain a high level of activity.

**Fig. 5:**
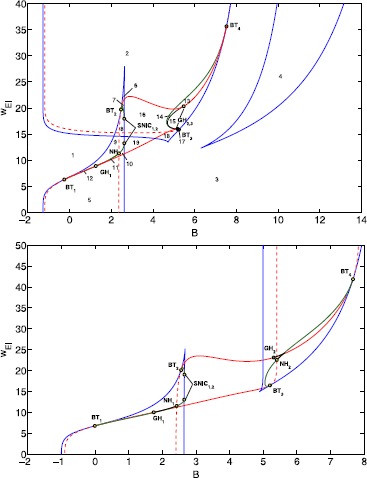
Bifurcation diagrams for Gaussian (*top*) and sigmoid (*bottom*) activation function. The background input *B* and the coupling parameter $w_{EI}$ are varied. Other parameters as in Sect. 2. Bifurcation curves are indicated with color: saddle-node (*blue*), Hopf (*red*), limit point of cycles (*black*), homoclinic to saddle (*green*). The red dashed line indicates a neutral saddle, which is not a bifurcation but here an LPC emerges from a homoclinic bifurcation. Note that our diagram for the sigmoid differs from [22] as we have modified the activation functions

The additional steady state is a robust feature that coexists with the normal dynamical repertoire of the Wilson–Cowan model with a sigmoid. To show this, consider the bifurcation diagram in the $(B,w_{EI})$-parameter plane as shown in Fig. 5. We have chosen to vary these parameters as this combination controls the level of activity of the populations and the strength of the feedback loop, and hence the dynamics, i.e. stable steady states and periodic oscillations. An earlier study [22] also presented a bifurcation analysis for the sigmoid case varying these parameters. Hence we can compare the two diagrams, where most bifurcation curves are similar. Our shift in thresholds $E_{\theta}$, $I_{\theta}$ results in a larger region with stable oscillations than in [22] for both Gaussian and sigmoid. For the Gaussian we see that there is an additional saddle-node bifurcation curve, not present for the sigmoid, which corresponds to the additional steady state. It is characterized by high values of $w_{EI}$ and to lower values of *B*, such that the excitatory population can drive the inhibitory population into depolarization block.

**Fig. 6:**
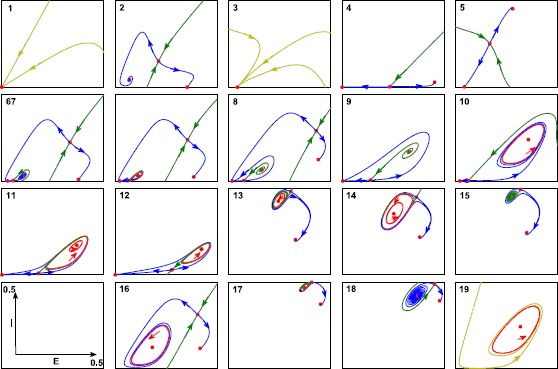
Phase portraits for Gaussian FRF. Characteristic phase portraits for all 19 regions for a single local population. Numbers correspond to parameter values in areas as in Fig. 5. *Red* indicates equilibrium or limit cycle, stable manifolds are *green*, unstable manifolds are *blue* and orbits *yellow*

For a complete understanding of the bifurcation diagram for the Gaussian case, we have generated characteristic phase portraits for all 19 parameter regions; see Fig. 6. Starting in region 1, we find a single low stable equilibrium. Crossing a saddle-node bifurcation to areas 2 or 5, two equilibria with high excitatory activity appear. Whereas in area 2 depolarization block plays a role, in area 5 the coupling is too low for depolarization block to occur and the inhibitory population is active too. Next, crossing saddle-node bifurcations to area 3, there is a single stable node again, while in area 4 we have three equilibria, one saddle, one with stable low activity and one with high excitatory and high inhibitory activity, different from the one in area 2. On the saddle-node bifurcation curves we find, in total, four Bogdanov–Takens (BT) bifurcations. From each BT-point a Hopf curve emerges and each of these ends up in another BT-point. Along a Hopf bifurcation we find degeneracies where the Hopf bifurcation changes from super- to subcritical. Here a limit point of cycle (LPC) bifurcation curve emerges that ends in a point where the saddle along a homoclinic curve is a neutral saddle (NH). The homoclinic curves either end in saddle-node homoclinics (SNIC) or connect to another BT-point. The parameter region for which we find stable oscillations, is made up of areas 7, 10, 11, 14, 16, 19, and it is delineated by Hopf, homoclinic, LPC and SNIC bifurcation curves. All other transitions involve unstable invariant sets, and therefore we do not discuss them. Phase portraits in areas 1&3, 2&4&5&18, 12&13&17, 9&15, 10&16 and 11&14 are structurally equivalent, but are shown for completeness as the amount of inhibitory activity varies.

### Two Excitatory Coupled E-I Pairs

**Fig. 7:**
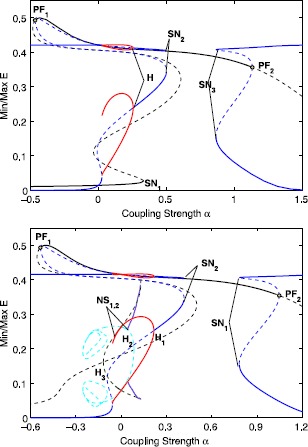
One parameter bifurcation diagram for $B=2.45$ (*top*) and $B=3.0$ (*bottom*). Colors indicate solution types: symmetric (*black*) and asymmetric (*blue*) steady states and symmetric (*green*) and in-phase asymmetric (*red*) and anti-phase asymmetric (*light-blue*) oscillations. Bifurcation labels are *SN* for saddle-node, *PF* for pitchfork, and *H* for Hopf. For the asymmetric branches, the upper part corresponds to one population, say $E_{1}$, and then the lower part corresponds to the other population $E_{2}$. The extremal values of *E* for quasi-periodic oscillations are indicated by purple lines. *Thick lines* indicate stable solution branches, *thin dashed lines* correspond to unstable branches

Here we discuss the dynamical behavior for two coupled populations. Above we have discussed the bifurcation diagram for a single excitatory-inhibitory pair. We fix $w_{EI}=18$ from now on to ensure the additional steady states exists. We choose two representative values for *B* with different dynamics for a single pair. For $B=2.45$, we have two stable equilibria, one with high and the other with low excitatory activity. For $B=3$, the stable high activity equilibrium remains, but the other attractor is a stable oscillation. This corresponds to areas 8 and 16 in Fig. 6. For both values, we construct a one parameter diagram by varying *α* the coupling strength between excitatory populations; see Fig. 7. Here, for continuity, we also show what happens for negative *α*, although this is not relevant neurophysiologically. Also, we omit several bifurcations and unstable branches that would obscure the presentation. The complete diagrams can be found in the supplementary material.

**Fig. 8:**
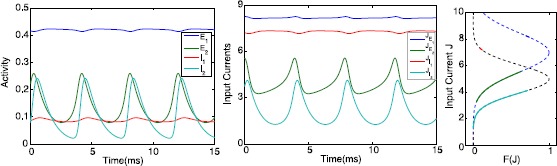
Dynamics of the asymmetric in-phase oscillation. *Left*: Time-series of the activity of excitatory and inhibitory populations of the asymmetric in-phase oscillation for $B=2.3$ and $\alpha=0.1$. *Middle*: corresponding time-series of the input currents. *Right*: The dynamical range of input currents *J* along the excitatory (*blue*) and inhibitory (*black*) activation functions

Starting from $\alpha=0$ with $B=2.45$, we first follow the symmetric low steady state (black line) around $E=0.01$. Increasing *α*, it becomes unstable at a saddle-node $\mathit{SN}_{1}$ at $\alpha\approx0.33$. Following the symmetric branch, we get to the high steady state. It is stable between the two pitchfork bifurcations $\mathit{PF}_{1}$ at $\alpha\approx -0.467$ and $\mathit{PF}_{2}$ at $\alpha\approx1.13$. From $\mathit{PF}_{2}$ an unstable asymmetric steady state emerges, which becomes stable at a saddle-node bifurcation $\mathit{SN}_{3}$ at $\alpha\approx0.86$. For this stable asymmetric equilibrium with high coupling strength, one excitatory population drives the other into depolarization block. The asymmetric steady state near $\mathit{PF}_{1}$ is unstable, but becomes stable at a saddle-node $\mathit{SN}_{2}$ at $\alpha\approx0.502$. Then decreasing *α* from this saddle-node, we encounter a supercritical Hopf bifurcation *H* at $\alpha\approx0.255$. Here we find a solution branch of stable asymmetric in-phase limit cycles which ends in a saddle-node homoclinic bifurcation. The periodic orbit has small amplitude fluctuations (maximal amplitude ≈0.015) with high excitatory activity in one population. The amplitude in the other population is much larger as large as 0.2; see also Fig. 8 for a time-series. For this branch we have also plotted the range of input currents $J_{E,I}$ along the activation functions. It shows for population 1 that the input current is quite high but of small amplitude. For population 2 the values are lower but the ranges are larger. Since the EEG does not capture the spikes and filters out the DC-component, in an experiment this would give the counter-intuitive result of high spiking activity accompanied with low amplitude EEG output, whereas, in contrast, its neighbor has low spiking activity but a markedly higher amplitude EEG output.

**Fig. 9:**
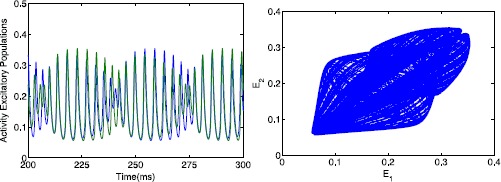
Quasi-periodic behavior. *Left*: Time-series of the activity of excitatory and inhibitory populations of the quasi-periodic solution for $B=3$ and $\alpha =0.115$. *Right*: Projection to the $(E_{1},E_{2})$-plane. When the torus is close to the symmetric oscillation, this is close to the diagonal

Next we consider the bifurcation diagram for $B=3$; see Fig. 7(bottom). Regarding the steady states it is quite similar. The high symmetric steady state is still stable between $\mathit{PF}_{1}$ and $\mathit{PF}_{2}$, but the symmetric low steady state is always unstable. The oscillations on the other hand are quite different. The in-phase asymmetric oscillation is similar starting from $H_{1}$, but now there is also a stable anti-phase solution. This periodic orbit emerges from a Hopf bifurcation $H_{2}$ of the symmetric steady state at $\alpha \approx0.083$. This oscillation is stable for $0<\alpha<0.043$, where at $\alpha\approx0.043$ a supercritical Neimark–Sacker bifurcation occurs. There is also a Hopf bifurcation $H_{3}$ leading to symmetric limit cycles. For the quasi-periodic attractor, we determined the minimal and maximal values of the excitatory activity using simulations; see Fig. 9. These simulations suggest that the torus first evolves around the anti-phase solution, then escapes to the symmetric oscillation for some time and returns near the anti-phase solution, and so on. Increasing *α*, the torus ends in some global bifurcation where it jumps to the asymmetric in-phase oscillation.

### Spatial Dynamics

**Fig. 10:**
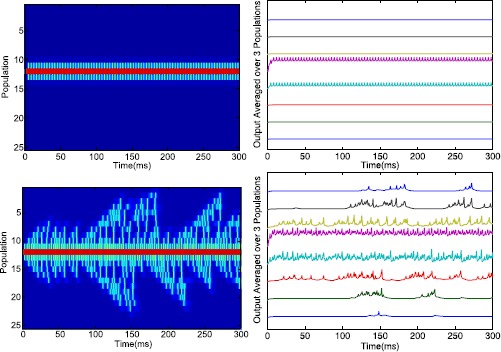
Local and global activity. *Left*: Activity of excitatory populations after stimulation between $1\leq t\leq5$ with $B=2.3$ (*top*) and $B=2.45$ (*bottom*) and $\alpha =0.1$. *Right*: Model EEG output

Our analysis revealed a stable asymmetric in-phase oscillation for two populations. Here we discuss the consequences for a larger network with 25 populations. We set $B=2.3$ and $\alpha=0.1$ and put all the populations in a stable low activity equilibrium. Between $t=1$ and $t=5$ we give an additional stimulus to $E_{12}$, i.e. we set $B_{12}\rightarrow B_{12}+2$, and not in the center to keep it asymmetric. This population then switches to the high activity steady state and forces its neighbors into an oscillatory mode similar to the asymmetric in-phase oscillation; see Fig. 10. Note that only the direct neighbors are driven and that the activity of other cells remains very low. Hence, the oscillation stays localized. Next, we increase the background activity to $B=2.45$ and repeat the simulation and see that the oscillations can spread. Every so many cycles three or more populations are also recruited into an oscillatory mode. Such emitted waves end when it reaches the boundary or when several populations are active simultaneously as occurs around $t=152$ or $t=183$. So, for this value of *B*, the activity does not stay localized and one population continuously drives the whole network.

**Fig. 11:**
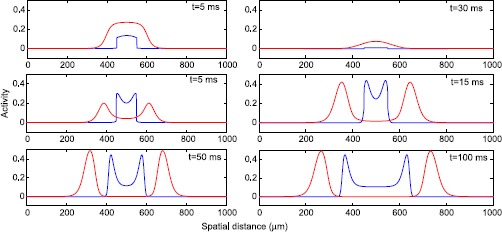
Propagation. *Top row*: Excitatory (*blue*) and inhibitory (*red*) activity with sigmoidal population activation function. Activity is extinguished by 100 ms. No propagation is present. *Middle row*: Population activities with Gaussian firing rate function. Here, a traveling wave pulse forms and begins to propagate. *Bottom row*: same as middle but at later times. The traveling wave continues to propagate until it dies at the boundary. The wavespeed is approximately 1 mm/s. Parameters are the same in each plot

Finally, we simulate the spatially continuous model; see Fig. 11. On the top row, a sigmoid firing rate function produces transient behavior but no traveling pulse. The middle and bottom rows show a propagating wave associated with the introduction of a Gaussian activation function. Here, we can clearly see a wave originating in the middle and propagating to the edges. The excitatory activity provides sufficient input to the inhibitory neurons to drive them into depolarization block and the inhibitory activity is not strong enough to keep the activity localized. Thus, we may conclude that our formalism provides a mechanism for dynamic disinhibition arising from depolarization block which the sigmoid firing rate function has not been able to reproduce. One more thing to notice is that, while the input is only to the excitatory neurons, the excitatory pulse of excitation lags behind inhibition, a finding consistent with detailed recordings of epileptiform activity [8]. In [6] the speed of the wave was estimated around 0.8 mm/s. We varied the strength of the excitatory coupling to match the wave speed in the model with this experimental value.

## Discussion

In this paper, we have investigated the dynamics of a neural network governed by the Wilson–Cowan equations. In particular, we have chosen a different Gaussian activation function, rather than the default sigmoid. We have found the existence of an additional high excitatory steady state due to the Gaussian and we focused on its consequences for network dynamics. Many of the other attractors in our bifurcation analysis have been discussed in earlier studies [22, 23]. With multiple local populations connected, the high activity provided strong drive to the surrounding populations resulting in breather-like dynamics. Beyond critical parameter values, the activity could spread through the whole network.

The Gaussian activation function was motivated by observations of ictal activity recorded using a Utah array. An experimental activation function could be determined using the low-frequency component of the LFP as a measure of synaptic input, and the high-frequency component as spike output. For some cases this showed a nonmonotone relationship suggesting the choice for the Gaussian. This relationship reflects multiple sources and also represents inhibitory and excitatory cells. As cortical networks consist of 80% large excitatory neurons and 20% small inhibitory interneurons, one would interpret the graph in Fig. 1C as predominantly originating from the excitatory population. The experimental curve in Fig. 1C suggests that beyond the maximum a plateau is reached. It could be that some of the large excitatory cells still exhibit a sigmoidal relationship at these high L-LFP levels. Then it is not unreasonable to assume the inhibitory cells exhibit depolarization block even earlier. For simplicity, we have modeled the activation functions for both populations as a Gaussian which approaches zero for high input, but the input may not even achieve such levels. Indeed, in our simulations the input never went far beyond the maximum for *E*. We then found that, for the standard choice of the model parameters, there is an additional stable equilibrium with high excitatory and low inhibitory activity. This steady state coexists with the typical low activity equilibrium and oscillations. For this equilibrium to exist, the precise form of the activation function is not important as long as the inhibitory FRF has a maximum and then drops sufficiently for high input, e.g. due to depolarization block. Indeed Fig. 4 shows that the shape of the inhibitory nullcline is most crucial for generating the additional steady state.

The bifurcation analysis for two coupled local populations showed multiple asymmetric stable attractors. Depending on the value of the coupling parameter *α*, either low or high, one population has high and the other low excitatory steady state activity. For an intermediate range of coupling strengths, there are also stable oscillations where one pair has small fluctuations around the higher steady state, while the other has large amplitude oscillations around a lower steady state. In a network with more pairs, we found that these oscillatory solutions can act as a driver towards neighboring populations. We should remark that the activity and the synaptic input differ quite a bit in their time course. Indeed cells with high excitatory activity receive a high synaptic input of relatively constant amplitude. The nearby populations have oscillatory activity with lower amplitude, but the amplitude of the synaptic input currents varies much more.

This is consistent with the recent proposal that an epileptic focus consists of a core and penumbra [4]. The border of the core has a lot of spike activity, whereas the surrounding has less spiking activity. On the other hand, LFP recordings representing synaptic activity, show the reverse situation with high amplitude signals in the penumbra and low amplitudes in the core. In addition, recent experimental recordings of seizures showed that spikes from inhibitory cells were nearly absent, but still many spikes from excitatory cells were observed [8]. If the core receives high levels of input with relatively little fluctuations, so that the LFP with the DC-offset filtered away shows little signal, the inhibitory cells may actually experience a depolarization block. Subsequently, the inhibitory neurons can no longer veto ongoing epileptiform activity similar to observations in experimental seizures [4]. In our model, we find for our new asymmetric attractors large model-EEG signals in the penumbra and much smaller in the core. Hence, our model supports the idea of core and penumbra of an epileptic focus with different levels of activity corresponding to large and small LFP amplitudes. Also our model predicts that the DC-component of LFP would show interesting shifts in the core. Recent work by Jirsa [3] also argues that the DC-component during a seizure is quite different from normal conditions. We have only shown data recorded within the core. We have examined the activity of areas within the penumbra, but the dynamic range was so small that we could not interpret this data. Hence, it would be interesting to determine in another way the activation functions outside the areas with epileptiform activity.

In our spatially continuous model, we showed that such a seizure can spread as a traveling front where inhibition is leading; see Fig. 11. In contrast, the recording in Fig. 1A has been considered in a recent modeling paper [28] using the same Wilson–Cowan model and a sigmoidal FRF. In that study, a parameter change was needed to decrease inhibition, whereas our use of a Gaussian FRF leads dynamically to decreased inhibition. Also their simulations suggest excitatory activity is leading at the front. In contrast, our simulations agree with the identification of the inhibitory spikes at the front [4, 8]. There is also an experimental seizure model where a subset of the inhibitory cells enter depolarization block during epileptiform activity [29]. Such experimentally well controlled settings might allow one to observe distinct neural populations during propagating seizures. In our model, the activity settles to the high steady state at the rear of the traveling front. A different dynamical mechanism for the propagation of epileptiform activity has been considered in [30] similarly modeled as in [28]. Their epileptiform activity invades surrounding tissue also as a traveling wave front, with multiple pulses emitted from a spatially homogeneously oscillating core. This oscillating core expands slower than the front. In this paper we focus on the front, but it would be interesting to consider the rear of the front in future work. We note that we only simulated our spatially continuous model using insights from the coupled populations. By approximating the activation function as a product of Heaviside-step functions, i.e. a blockpulse, we expect that it is possible to find implicit equations for the various phases of the traveling front and the speed using techniques as in [14]. This could elucidate the range of thresholds for depolarization block where our traveling front exists.

We do not attempt to argue that our model describes transitions between normal and ictal activity. As in many other modeling studies there can be exogeneous parameter transitions causing these changes [31–33]. The most influential parameters are the background input *B* and the local connection $w_{EI}$. Then already for medium coupling strength *α*, rich multi-stable asymmetric dynamics appears. We have also carried out experiments with noisy input. These show that the low activity steady state can escape to normal oscillatory behavior and then can further transition to the high activity steady state depending on the noise amplitude. We found that switches from low to oscillatory activity and vice versa can occur. Once the activity jumps to the high activity branch, the dynamics can only return to low activity levels if *B* or $w_{EI}$ is decreased substantially. Rather than changing a parameter artificially, the return to baseline may also be achieved by incorporating additional mechanisms such as energy consumption [34], de-inactivation of ion channels [35]. These act on a timescale from seconds to minutes and may be important to describe late phases of seizure activity.

## Electronic Supplementary Material

Full one parameter bifurcation diagrams. (PDF 106 KB)
