# Supplementary material for: Modeling Focal Epileptic Activity in the Wilson–Cowan Model with Depolarization Block
Source: J Math Neurosci. 2015 Mar 27;5:7. doi: 10.1186/s13408-015-0019-4 (PMC4385301; doi:10.1186/s13408-015-0019-4)
Supplement: Supplementary file 1 — Full one parameter bifurcation diagrams. (PDF 106 KB) [file 13408_2015_19_MOESM1_ESM.pdf]

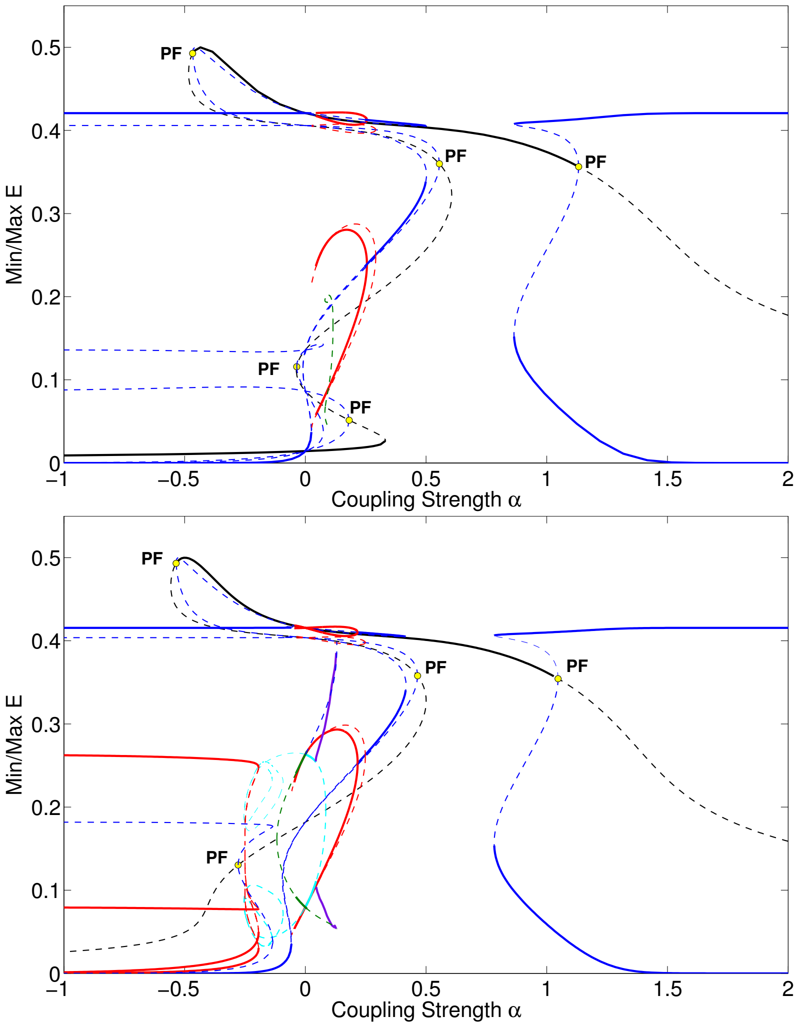

Figure 1 Full one parameter bifurcation diagrams for $B = 2.45$ (top) and $B = 3.0$ (bottom). Colors indicate solution types: symmetric (black) and asymmetric (blue) steady states and symmetric (green) and in-phase asymmetric (red) and anti-phase asymmetric (light-blue) oscillations. Bifurcation labels are SN for saddle-node, PF for pitchfork and H for Hopf. For the asymmetric branches, the upper part corresponds to one population, say E_1, and then the lower part corresponds to the other population E_2. The extremal values of E_1 for quasi-periodic oscillations are indicated by purple lines. Thick lines indicate stable solution branches, thin dashed lines correspond to unstable branches.
